# Antimicrobial Activity and Stability of Short and Long Based Arachnid Synthetic Peptides in the Presence of Commercial Antibiotics

**DOI:** 10.3390/molecules21020225

**Published:** 2016-02-17

**Authors:** Ivan Arenas, Elba Villegas, Oliver Walls, Humberto Barrios, Ramon Rodríguez, Gerardo Corzo

**Affiliations:** 1 Departamento de Medicina Molecular y Bioprocesos, Instituto de Biotecnología, Universidad Nacional Autónoma de México, UNAM. Apartado Postal 510-3, Cuernavaca, Morelos 62250, Mexico; arsoivan@gmail.com; 2 Centro de Investigación en Biotecnología, Universidad Autónoma del Estado de Morelos, Av. Universidad 2001, Cuernavaca, Morelos 62210, Mexico; elbav@uaem.mx; 3 Laboratorios Liomont SA de CV, Adolfo López Mateos 68, Cuajimalpa, Cuajimalpa de Morelos, México City 05000, Mexico; owalls@liomont.com.mx (O.W.); rrodrigu@liomont.com.mx (R.R.); 4 Instituto de Salud Pública, Av. Universidad 655, Santa María Ahuacatitlán, Cuernavaca, Morelos 62100, Mexico; humberto.barrios@insp.mx

**Keywords:** antibiotic, antimicrobial peptide, peptide, bacteria

## Abstract

Four antimicrobial peptides (AMPs) named Pin2[G], Pin2[14], P18K and FA1 were chemically synthesized and purified. The four peptides were evaluated in the presence of eight commercial antibiotics against four microorganisms of medical importance: *Escherichia coli*, *Staphylococcus aureus*, *Pseudomonas aeruginosa*, and *Klebsiella pneumoniae*. The commercial antibiotics used were amoxicillin, azithromycin, ceftriaxone, gentamicin, levofloxacin, sulfamethoxazole, trimethoprim and vancomycin. The best AMP against *P. aeruginosa* was the peptide FA1, and the best AMP against *S. aureus* was Pin2[G]. Both FA1 and Pin2[G] were efficient against *E. coli*, but they were not effective against *K. pneumoniae*. As *K. pneumoniae* was resistant to most of the commercial antibiotics, combinations of the AMPs FA1 and Pin2[G] were prepared with these antibiotics. According to the fractional inhibitory concentration (FIC) index, the best antimicrobial combinations were obtained with concomitant applications of mixtures of FA1 with levofloxacin and sulfamethoxazole. However, combinations of FA1 or Pin2[G] with other antibiotics showed that total inhibitory effect of the combinations were greater than the sum of the individual effects of either the antimicrobial peptide or the antibiotic. We also evaluated the stability of the AMPs. The AMP Pin2[G] manifested the best performance in saline buffer, in supernatants of bacterial growth and in human blood plasma. Nevertheless, all AMPs were cleaved using endoproteolytic enzymes. These data show advantages and disadvantages of AMPs for potential clinical treatments of bacterial infections, using them in conjunction with commercial antibiotics.

## 1. Introduction

Since the development of penicillin in the 1940s, the synthesis and use of different antibiotics has had an important impact on human health; however, the emergence of resistant bacteria has made bacterial infections increasingly difficult to treat with available antibiotics. Thus, one of the major challenges to today’s world health systems is the appearance and spread of resistance to antibiotics. Many pathogenic bacteria that were once sensitive to conventional antibiotics are now multidrug resistant, reducing the therapeutic value of these antibiotics [1]. This has encouraged a search for leads indicating novel antibiotics among natural compounds that are able to bypass the known mechanisms for drug resistance [2]. The antimicrobial peptides (AMPs) may constitute an alternative for this, serving directly or in combination with classical antibiotics [3,4]. However, two critical disadvantages of these antimicrobial peptides are their size and their poor stability in the presence of proteolytic enzymes. Therefore, the aim of this work was to test four designed synthetic peptides, based on native arachnid peptides of different sizes, and to observe their stability under different conditions as well as their antimicrobial capacity in the absence and presence of existing antibiotics.

## 2. Results

### 2.1. Isolation and Sequence Analysis

**Figure 1:**
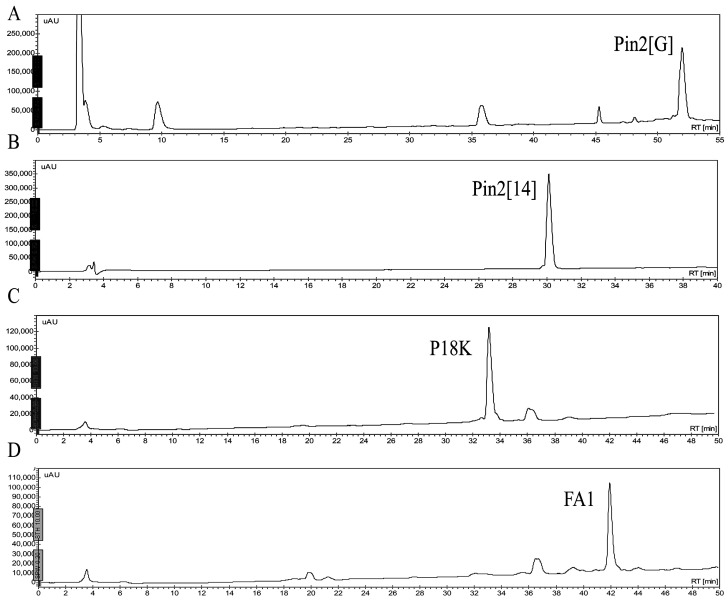
Reverse phase separation of synthetic peptides. They were separated using an analytical reverse phase C_18_ column, at flow of 1 mL/min, with linear gradients from 0 to 60 of acetonitrile containing 0.1% of trifluoroacetic acid, in 60 min. (**A**) Pin2[G]; (**B**) Pin2[14]; (**C**) P18K; and (**D**) FA1.

Four peptides were chemically synthesized and purified by reverse phase HPLC (Figure 1). The retention time of each peptide, under the same conditions, showed that their hydrophobic character ranged from less to more hydrophobic; Pin2[14] < P18K < FA1 < Pin2[G]. The experimental data concurred with the calculated GRAVY (Table 1). Other theoretical parameters of these four peptides included their average molecular flexibility (AMF), hydrophobic moment (µH) and net charge (Q) (Table 1). The hydrophobic moment of an alpha-helical peptide provides an indication of its amphipathicity; so, a value of 0.5 indicates that the peptide has about 50% of the maximum possible amphipathicity. Here, all four peptides are predicted to carry between 41% and 57% amphipathicity. Likewise, the retention times provided a reference for observing their hydrophobicity and their stability after exposition to saline buffer, bacterial supernatants, human blood and proteolytic enzymes.

### 2.2. Antimicrobial Activities of Peptides

**Figure 2:**
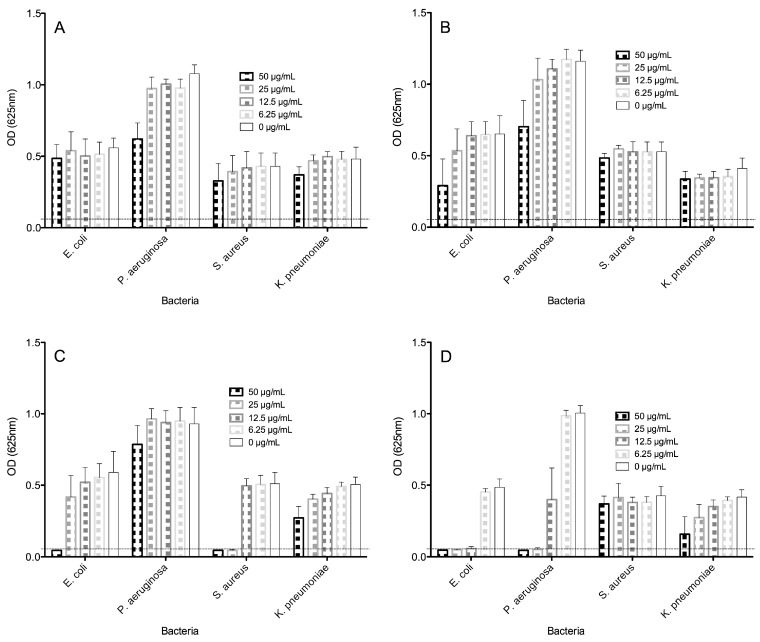
Antimicrobial activities of peptides against bacteria. Different concentrations of Antimicrobial peptides (AMPs) were challenged against the growth of *E. coli* ATCC 25922, *S. aureus* ATCC 25923, *Pseudomonas aeruginosa* ATCC 27853 and *Klebsiella pneumoniae* ST258 in Muller-Hinton broth (*n* = 8). (**A**) Pin2[14]; (**B**) P18K; (**C**) Pin2[G] and (**D**) FA1. Bacteria-free well absorbances are indicated by a dotted line.

All four peptides were first assayed for antimicrobial activity towards *Escherichia coli* ATCC 25922, *Staphylococcus aureus* ATCC 25923, *Pseudomonas aeruginosa* ATCC 27853 and *Klebsiella pneumoniae* ST258 (Figure 2). Here, the peptides were tested in the presence of antibiotics using the microdilution assay broth, which is a more astringent assay than that of the agar-diffusion assay, mainly because the peptides and the proteolytic enzymes produced by the bacterial cells being tested are in a homogenous culture medium that is different to a restricted semi-solid agar medium.

Although there was no clear correlation between the molecular size, hydrophobicity and biological activity in the case of all four peptides tested, the largest and most hydrophobic peptides were the most effective against all four bacteria, as opposed to the shortest and least hydrophobic peptides.

This means that the largest peptides FA1 and Pin2[G] manifested the most effective antimicrobial activity towards all four bacteria tested, however, the peptide FA1 was the most active against Gram negative bacteria, and Pin2[G] was the most potent against the Gram positive *S. aureus*. Bacteria-free well absorbances are indicated by a dotted line in Figure 2; thus, the minimal inhibitory concentrations (MICs) for Pin2[14] were >50, >50, >50 and >50 µg/mL, for P18K were >50, >50, >50 and >50 µg/mL, for FA1 were 12.5, 25, >50 and >50 µg/mL and for Pin2[G] were 50, >50, 25 and >50 µg/mL for *E. coli*, *P. aeruginosa*, *S. aureus* and *K. pneumoniae*, respectively (Figure 2 and Table 2). Consequently, no MICs were found for Pin2[14] and P18K in the case of all four microorganisms tested. The most resistant bacteria was *K. pneumoniae* and relatively the most vulnerable was *E. coli.*

### 2.3. Antimicrobial Activities of Selected Antibiotics

**Figure 3:**
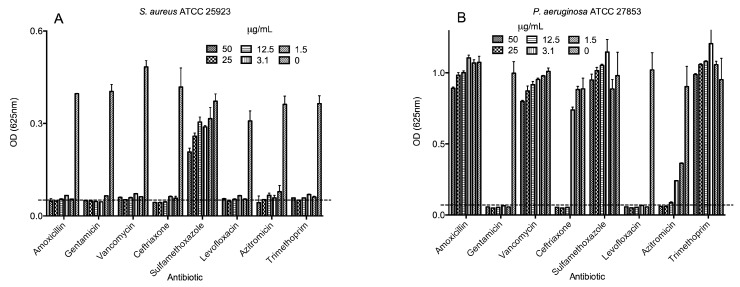

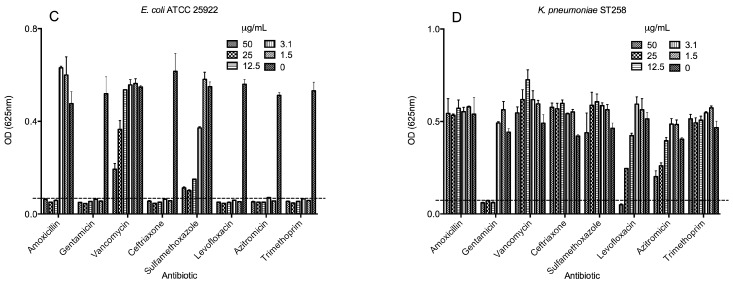
Dose-response activities of selected antibiotics against bacteria. The eight commercial antibiotics were tested against the growth of (**A**) *Staphylococcus aureus* ATCC 25923; (**B**) *Pseudomonas aeruginosa* ATCC 27853; (**C**) *Escherichia coli* ATCC 25922; and (**D**) *Klebsiella pneumoniae* ST258 in Muller-Hinton broth. Bacteria-free well absorbances are indicated by a dotted line.

Similarly, all eight antibiotics were assayed for antimicrobial activity towards *E. coli*, *S. aureus, P. aeruginosa* and *K. pneumoniae* (Figure 3). *S. aureus* was the most susceptible bacteria to the commercial antibiotics tested; it only showed resistance to sulfamethoxazole (Figure 3A). *P. aeruginosa* was resistant to amoxicillin, vancomycin, sulfamethoxazole and trimethoprim (Figure 3B). *E. coli* was the second most susceptible bacteria, with resistance to vancomycin and to some extent to sulfamethoxazole (Figure 3C). The most resistant bacteria were *K. pneumoniae*, which could grow in the presence of trimethoprim, sulfamethoxazole, azithromycin, amoxicillin, ceftriaxone and vancomycin (up to 50 µg/mL) and to some extent in the presence of levofloxacin (up to 25 µg/mL); however, *K. pneumoniae* was susceptible to gentamicin at the concentrations used (Figure 3D). Table 2 summarizes the MIC found for all four bacteria. Interestingly, all four bacteria were resistant to sulfamethoxazole, but all were susceptible to levofloxacin and gentamicin (except *K. pneumoniae*).

### 2.4. Biological Activities of Selected Peptides and Antibiotics

**Figure 4:**
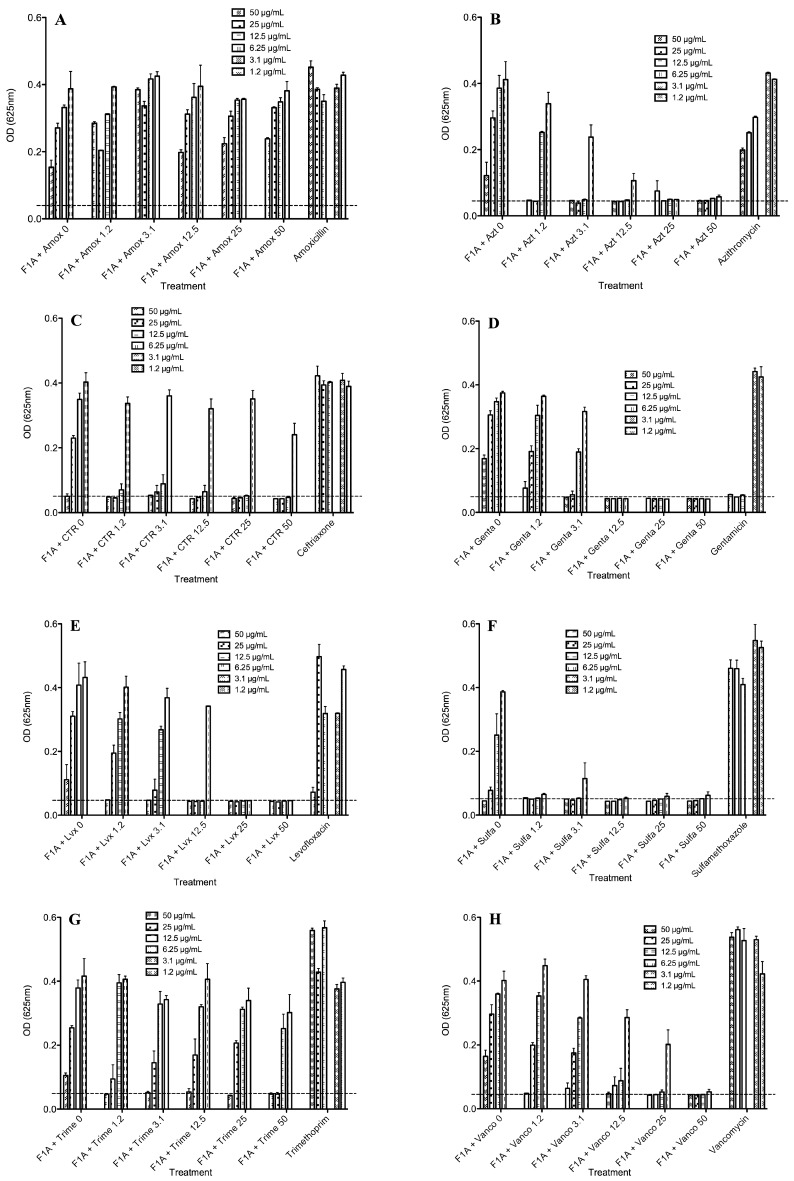
Antimicrobial activity of FA1 in the presence of eight different commercial antibiotics against *K. pneumoniae*. (**A**) Amoxicillin; (**B**) Azithromycin; (**C**) Ceftriaxone; (**D**) Gentamicin; (**E**) Levofloxacin; (**F**) Sulfamethoxazole; (**G**) Trimethoprim and (**H**) Vancomycin. Bacteria-free well absorbances are indicated by a dotted line.

**Figure 5:**
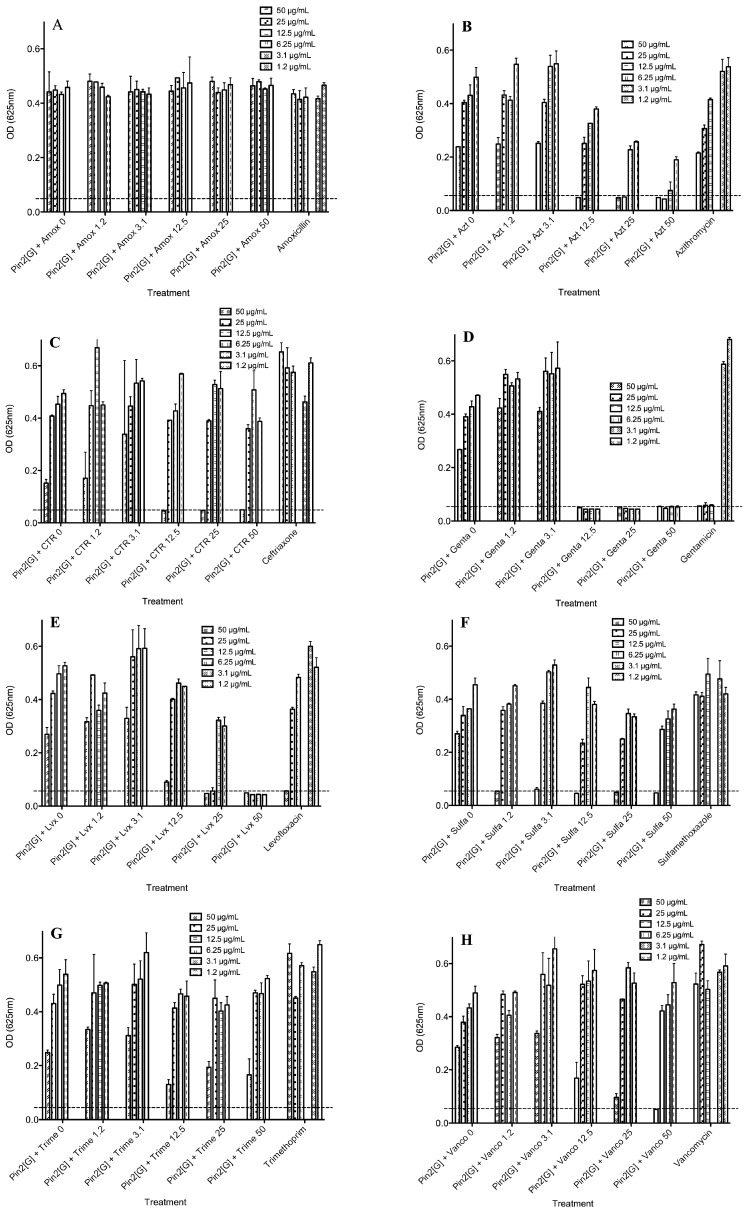
Antimicrobial activity of Pin2[G] in the presence of eight different commercial antibiotics against *K. pneumoniae*. (**A**) Amoxicillin; (**B**) Azithromycin; (**C**) Ceftriaxone; (**D**) Gentamicin; (**E**) Levofloxacin; (**F**) Sulfamethoxazole; (**G**) Trimethoprim and (**H**) Vancomycin. Bacteria-free well absorbances are indicated by a dotted line.

As *K. pneumoniae* was the most resistant bacteria to the AMPs and showed significant resistance to most of the antibiotics, combinations of the best AMPs; FA1 and Pin2[G], were combined with all eight antibiotics and tested against *K. pneumoniae* (Figure 4 and Figure 5). Figure 4 shows the antimicrobial activity of Pin2[G] in combination with the eight antibiotics. As observed in Figure 3D, *K. pneumoniae* was susceptible to levofloxacin and gentamicin; therefore, as expected the mixtures of the AMPs with these two antibiotics showed the best results. Furthermore, combinations of FA1 with trimethoprim, sulfamethoxazole, azithromycin, ceftriaxone and vancomycin improved the inhibition of *K. pneumoniae* to some extent (Figure 4). Likewise, the combinations of Pin2[G] with sulfamethoxazole, azithromycin, ceftriaxone and vancomycin somewhat improved the inhibition of this bacteria. However, higher concentrations of Pin2[G] were required, in order to observe greater inhibition effects (Figure 5). Table 3 summarizes the best concentrations for inhibiting the growth of *K. pneumoniae*. Also, the same table shows the FIC indexes, indicating the synergy, additive effects or antagonism of the mixtures used [9]. The best combinations of the peptide FA1 were with levofloxacin, and sulfamethoxazole, showing FIC indexes lower than 0.5. Furthermore, combinations of the peptide FA1 with azithromycin, ceftriaxone and vancomycin also demonstrated the inhibition of *K. pneumoniae* showing that the total effect of such mixtures was greater than the sum of the individual effects (here, FIC <1). Concerning the performance of the peptide Pin2[G] with antibiotics against *K. pneumoniae*, the best combinations of the peptide were with azithromycin and levofloxacin even though the FIC indexes were lower than 1 indicating an additive effect rather than a synergic one according to White *et al.* [9]. However, the combinations of Pin2[G] with azithromycin and levofloxacin clearly show that the effect of such mixtures is also greater than the sum of the individual effects. Interestingly, vancomycin, which is not very active against Gram-negative bacteria because it inhibits cell wall synthesis mainly in Gram-positive bacteria, inhibited the growth of *K. pneumoniae* in the presence of FA1 (Figure 4H).

### 2.5. Stability of Peptides in Saline Buffer, Human Blood Plasma and in the Presence of Enzymes

**Figure 6:**
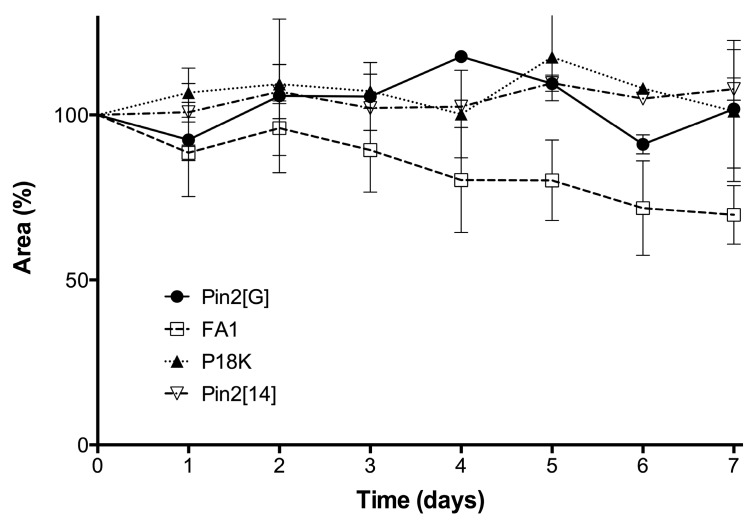
Stability of peptides under saline buffer. The AMPs were diluted in PBS (pH 7.4) and incubated a 37 °C for seven days.

**Figure 7:**
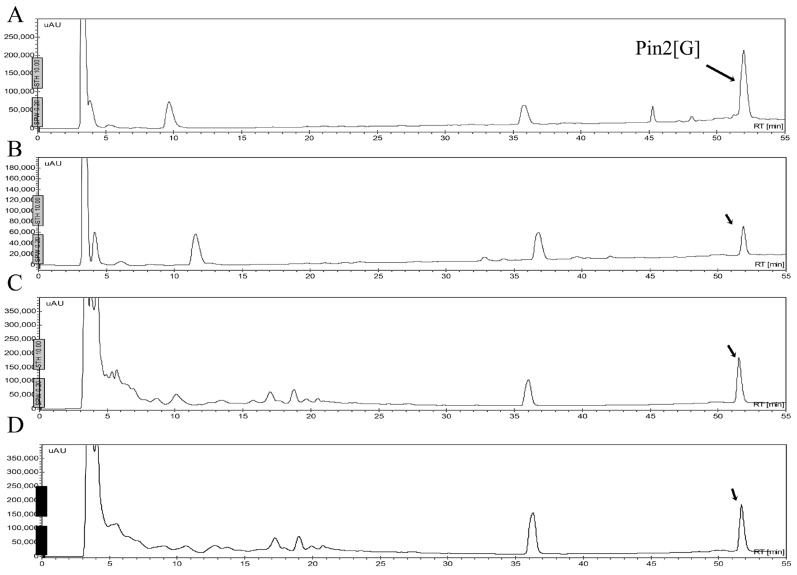
Stability of Pin2[G] in human blood plasma and bacterial supernatants. The peptide was either diluted in human blood or bacterial supernatants, incubated at 37 °C for 12 h and then extracted. (**A**) Pin2[G] was diluted with blood plasma, immediately extracted and separated by HPLC (control); (**B**) Pin2[G] was diluted with blood, incubated for 12 h at 37 °C, extracted and separated by HPLC; (**C**) Pin2[G] was diluted with *Staphylococcus aureus* supernatant, incubated for 12 h at 37 °C, extracted and separated by HPLC; and (**D**) Pin2[G] was diluted with *Pseudomonas aeruginosa* supernatant, incubated for 12 h at 37 °C, extracted and separated by HPLC. The arrows show the presence of Pin2[G].

**Figure 8:**
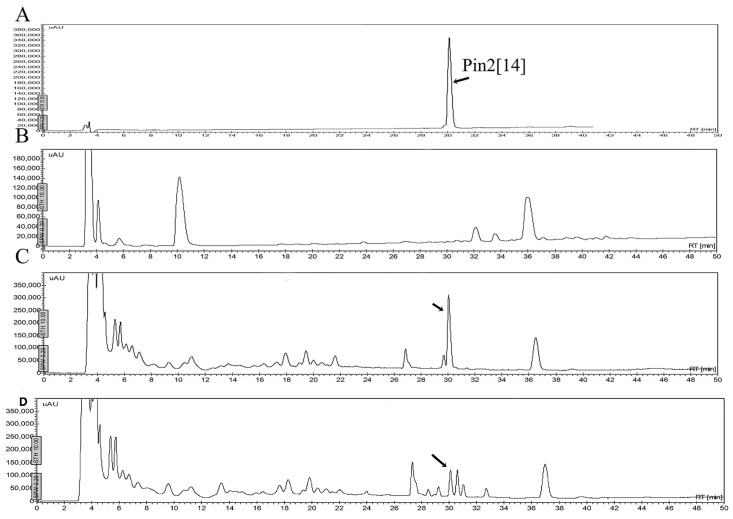
Stability of Pin14[G] in human blood plasma and bacterial supernatants. The peptide was either diluted in human blood or bacterial supernatants, incubated at 37 °C for 12 h and then extracted. (**A**) Pin14[G] was separated by HPLC (control); (**B**) Pin14[G] was diluted with blood, incubated for 12 h at 37 °C, extracted and separated by HPLC; (**C**) Pin14[G] was diluted with *Staphylococcus aureus* supernatant, incubated for 12 h at 37 °C, extracted and separated by HPLC; and (**D**) Pin14[G] was diluted with *Pseudomonas aeruginosa* supernatant, incubated for 12 h at 37 °C, extracted and separated by HPLC. The arrows show the presence of Pin14[G].

**Figure 9:**
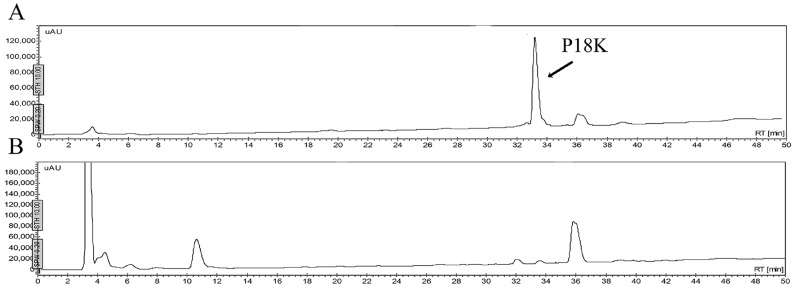

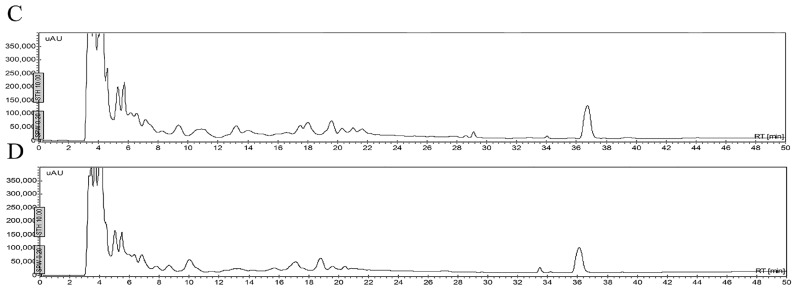
Stability of P18K in human blood plasma and bacterial supernatants. The peptide was either diluted in human blood or bacterial supernatants, incubated at 37 °C for 12 h and then extracted. (**A**) P18K was separated by HPLC (control); (**B**) P18K was diluted with blood, incubated for 12 h at 37 °C, extracted and separated by HPLC; (**C**) P18K was diluted with *Staphylococcus aureus* supernatant, incubated for 12 h at 37 °C, extracted and separated by HPLC; and (**D**) P18K was diluted with *Pseudomonas aeruginosa* supernatant, incubated for 12 h at 37 °C, extracted and separated by HPLC. The arrow shows the presence of P18K.

**Figure 10:**
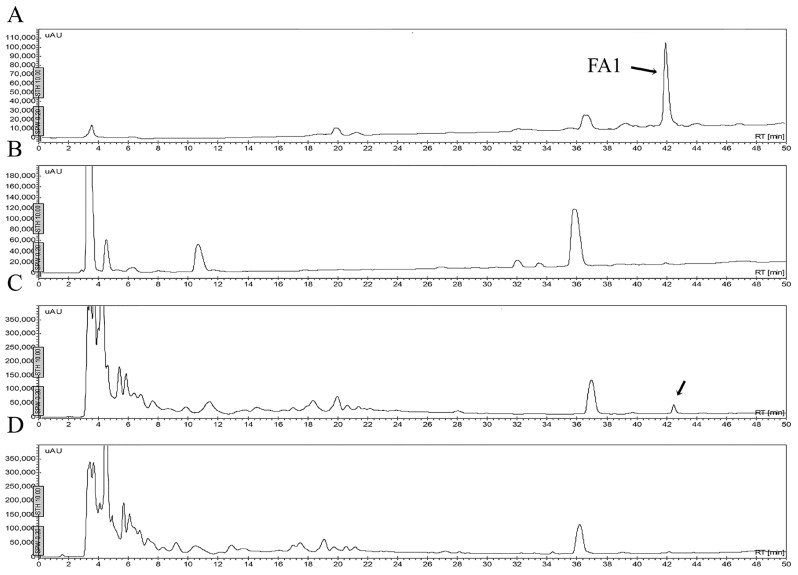
Stability of FA1 in human blood plasma and bacterial supernatants. The peptide was either diluted in human blood or bacterial supernatants, incubated at 37 °C for 12 h and then extracted. (**A**) FA1 was separated by HPLC (control); (**B**) FA1 was diluted with blood, incubated for 12 h at 37 °C, extracted and separated by HPLC; (**C**) FA1 was diluted with *Staphylococcus aureus* supernatant, incubated for 12 h at 37 °C, extracted and separated by HPLC; and (**D**) FA1 was diluted with *Pseudomonas aeruginosa* supernatant, incubated for 12 h at 37 °C, extracted and separated by HPLC. The arrow shows the presence of FA1.

One of the problems cited for using antimicrobial peptides refers to their stability in different fluids such as aqueous solutions, or in the presence of human blood plasma and/or in the presence of proteolytic enzymes. Figure 6 shows that the peptides may be stable in a buffered saline solution or in a sterile excipient able to carry these peptides as active ingredients. As observed, the peptide FA1 is less stable in saline solution during the time of experimentation. The decrement in concentration of FA1 in the saline solution may be due to absorption onto the vial surface. Although, the peptide FA1 is less hydrophobic than the peptide Pin2[G], FA1 may present larger hydrophobic clusters that interact with the vial surface. Figure 7, Figure 8, Figure 9 and Figure 10 show the stability of the AMPs in human blood plasma and in bacterial supernatants for a period of 12 h at 37 °C. Firstly, Pin2[G] was the peptide that maintained most of its integrity under these conditions. One quarter of its concentration was found in human plasma (Figure 7A,B), and it was not cleaved by the supernatants of *S. aureus* (Figure 7C) and *P. aeruginosa* (Figure 7D) after an incubation period of 12 h at 37 °C. Secondly, Pin2[14] was not found after extraction from human plasma (Figure 8A,B), and was not cleaved by supernatants of *S. aureus* (Figure 8C); neither was it found after treatment with *P. aeruginosa* supernatants (Figure 8D). Thirdly, neither was P18K obtained after extraction from human plasma (Figure 9A,B), as it was probably sequestered or cleaved by proteins in the supernatants of *S. aureus* (Figure 9C) and of *P. aeruginosa* (Figure 9D). Finally, FA1 was not found after extraction from human plasma (Figure 10A,B) and only a small amount of FA1 was obtained after treatment with supernatants from *S. aureus* (Figure 10C), but was not found after treatment with supernatants of *P. aeruginosa* (Figure 10D). Likewise, notably none of the AMPs, such as Pin2[14], P18K and FA1, were recovered after interaction with human plasma, even after the first few minutes of dilution, suggesting that these three peptides were strongly bound to proteins from human plasma.

**Figure 11:**
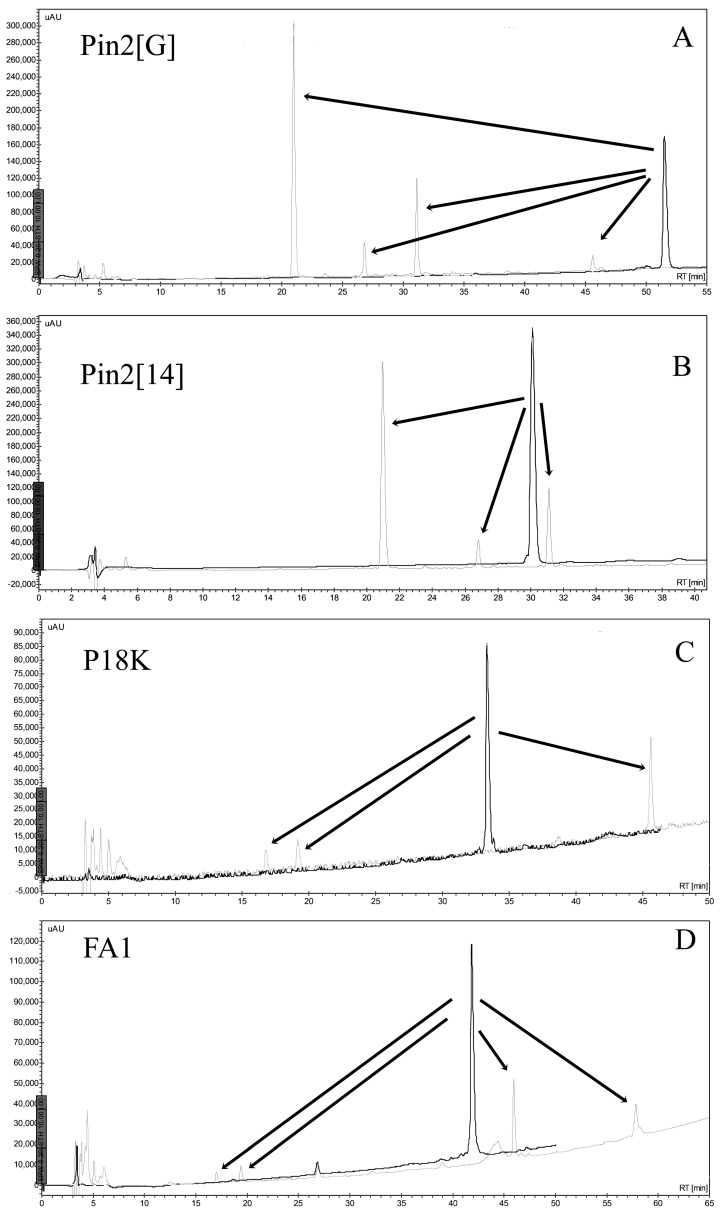
Stability of FA1 and Pin2[G] in the presence of elastase. The peptides were diluted in buffer and incubated at 37 °C for 12 h and then directly separated using reverse phase HPLC. (**A**) Pin2[G]; (**B**) Pin2[14]; (**C**) P18K; and (**D**) FA1.

The stability of AMPs in the presence of endoproteolytic enzymes was very poor. All AMPs were easily broken down in the presence of trypsin, elastase, chymotrypsin or pepsin. Figure 11 shows the HPLC profiles of the AMPs, including Pin2[G], Pin2[14], P18K and FA1, before and after their incubation in the presence of elastase. Elastase and elastase-type proteases are present in many different types of human cells, such as polynuclear leukocytes, neutrophils, smooth muscle cells, fibroblasts, blood platelets, and endothelial cells. Although human blood serum is rich in elastase inhibitors (mainly α_1_-proteinase inhibitor and α_2_-macroglobulin), human blood plasma is also involved in elastase-type endopeptidase activities [10]; so that this data reveals one more of the obstacles that would be faced by AMPs during a potential therapeutic application.

Although enzymes such as trypsin, chymotrypsin or pepsin are not present in blood serum, they represent enzymes that many pathogenic microorganisms release during infection. Nevertheless, the constant application of an antimicrobial peptide may compensate for any disadvantage related to enzymatic cleavage.

## 3. Discussion

In Gram negative bacteria, it is believed that most of the AMPs cross the outer bacterial membrane by a self-promoted uptake [11], similar to other cationic molecules. This means that AMPs displace divalent cations and because of their size cause distortion to the outer membrane structure. Upon contact with the inner membrane, AMPs become inserted, mainly because of the influence of the trans-membrane electrical potential gradient, which directs the cationic AMPs to the periplasm and then into the cytoplasmic membrane [12]. Besides this, the amphiphilic characteristics of AMPs help them to cross the cell membranes, employing several already proposed mechanisms, which drive the entry of AMPs through the lipid bilayer. Although the mechanism of action is not completely clear, they may use more than one action of mechanism depending on the nature of the AMP and the composition of the bacterial cell membrane. Mechanisms include transient or static pore formation and detergent-like solubilization [13].

Likewise, even though there is no experimental evidence indicating a synergistic self-promoted uptake of both AMPs and antibiotics, there is experimental proof that combinations of both AMPs and antibiotics are more microbicidal than the sum of the individual effects of either the AMP or the antibiotic. This implies they may cross the outer bacterial membrane by a self-promoted uptake, and then cause a cooperative cell membrane permeabilization. In this context, it is known that some AMPs have the ability to translocate across the biological membrane of bacterial cell membranes and even translocate cargo molecules into the cytoplasm [14]. Here, increasing concentrations of antimicrobial peptides in the presence of increasing concentrations of commercial antibiotics were investigated. However, increasing concentrations of antimicrobial peptides may imply a significant increase in the cost of the final product, as peptide synthesis is an expensive procedure. The selection of commercial antibiotics was based on their different structures and action mechanisms. Practically, all varieties of commercial antibiotics are represented here. In this sense, levofloxacin is a broad-spectrum antibiotic that is active against both Gram-positive and Gram-negative bacteria. It inhibits topoisomerases, which are enzymes important for DNA cell division. Trimethoprim inhibits dihydrofolate synthetase, an enzyme involved in the metabolic pathway to produce folate. Sulfamethoxazole inhibits the normal bacterial utilization of para-aminobenzoic acid for the synthesis of folic acid, an important metabolite in DNA synthesis. Azithromycin binds to the 50S subunit of the 70S bacterial ribosomes, thus inhibiting protein synthesis in bacterial cells. Amoxicillin acts by inhibiting the synthesis of bacterial cell walls, so that it specifically inhibits cross-linkage between the linear peptidoglycan polymer chains that make up a major component of the cell walls of both Gram-positive and Gram-negative bacteria. Similar to other cephalosporins, ceftriaxone inhibits bacterial cell wall synthesis, as it binds to the penicillin-binding proteins. Like other aminoglycosides, gentamicin irreversibly binds the 30S subunit of the bacterial ribosome, interrupting protein synthesis, and, finally, vancomycin inhibits cell wall synthesis mainly in Gram-positive bacteria; however, it is not active against Gram-negative bacteria.

Concerning AMPs, several physicochemical characteristics have been associated with the biological activity of them. Those that are relevant include hydrophobicity, the helical hydrophobic moment, as well as the size and the net charge of the peptide [13,15]. However, no particular value relating to any of these characteristics was solely sufficient to correlate with the antimicrobial activity of the AMPs tested. In this case, however, the larger peptides FA1 and Pin2[G] were the AMPs that manifested optimum biological performance. Nevertheless, a fine balance in terms of physicochemical properties may define a successful AMP, *i.e.*, one with high antimicrobial activity, low cytotoxicity and *in vivo* stability. This last characteristic is possibly the main disadvantage in the case of AMPs, hindering their use as successful therapeutic drugs. The results of this work therefore demonstrate that although AMPs may act synergistically or cooperatively with some conventional antibiotics, their *in vivo* stabilities currently prohibit them from offering real therapeutic value. In this case, FA1 was the peptide that demonstrated the best performance against *K. pneumoniae* and *S. aureus*, and Pin2[G] showed the best performance against *S. aureus*, but the possibility of using them systemically *in vivo* is uncertain. For example, the hemolytic activity of Pin2[14], P18K, FA1 and Pin2[G] was found to be 418, 923, 665 and 1.4 µM, respectively [16,17]. As Pin2[G] is more hemolytic than FA1, which implies a limited therapeutic window for systemic use of Pin2[G], thus confining its potential applicability to topical formulations. Pin2[G] may therefore be used for the treatment of dermal infections. In contrast to Pin2[G], FA1 is much less hemolytic, implying that it has a greater therapeutic window and making it suitable for use against pathogenic bacteria in soft tissue infections. However, the performance of FA1 in the presence of saline buffer or human plasma may represent a deficiency concerning its use as a therapeutic. Even though the two AMPs (Pin2[G] and FA1) manifest the best antimicrobial performances in *in vitro* conditions, they also present chemical deficiencies such as vulnerability to endoproteases (all four AMPs), bacterial secretions (Pin2[14], P18K and FA1), saline buffer (FA1) and stability in human blood plasma (Pin2[14], P18K and FA1). As mentioned previously, the biological performance of FA1 in *in vitro* assays appears to be better than that of the other three peptides, but its deficiencies in the presence of biological fluids are similar to those of the peptide Pin2[G]. For instance, the peptide Pin2[G] is more stable in blood plasma and on bacterial supernatants, so it can be used to cure dermal injuries. Likewise, the weakness of FA1 and Pin2[G] when they encounter proteolytic enzymes can be overcome by replacing key amino acidic residues to avoid this enzymatic cleavage. The use of d- or l-beta-amino acids may improve their stability in the presence of endoproteolytic enzymes [18]. Furthermore, it is unlikely that FA1 and Pin2[G] represent unique antimicrobial peptides because neither of them present exceptional qualities; however, by testing and revealing the best qualities of an antimicrobial peptide, it may be possible to design an AMP with less physicochemical deficiencies, which can be used as a therapeutic agent. This may be much improved if it can then be combined with available conventional antibiotics, in order to potentiate their antimicrobial capabilities in unison.

Although discoveries of novel antibiotic structures have been limited, the search for better antibiotics and antimicrobials continues. Meanwhile, the proliferation of resistant bacteria is escalating. One of the immediate solutions for contending with resistant bacteria has been the combination of conventional antibiotics; thus, mixtures of amoxicillin and azithromycin are currently available in the pharmaceutical market. Similarly, mixtures of novel antimicrobial agents with available commercial antibiotics may be crucial for limiting the spread of resistant bacterial strains. Additionally, the use of peptidic antimicrobial agents in combination may boost the use of inexpensive antibiotics such as sulfamethoxazole, or possibly open up markets for specific antibiotics in combination with cephalosporins. Though, the mechanism of action of AMPs in combination with antibiotics remains to be explored, these data may provide motivation for potential clinical treatments of bacterial infections using AMPs, in conjunction with commercial antibiotics.

## 4. Materials and Methods

### 4.1. Biologicals

The bacteria used were *Escherichia coli* ATCC 25922, *Staphylococcus aureus* ATCC 25923 and *Pseudomonas aeruginosa* ATCC 27853, which were purchased directly from the American Type Culture Collection through The Global Bioresource Center™ by UNAM. *Klebsiella pneumoniae* ST258 was kindly donated from the Institute of Public Health in Cuernavaca, Morelos, Mexico [5]. The antimicrobial peptides Pin2[G], Pin2[14], P18K and FA1 were chemically synthesized using the Fmoc methodology. Peptides Pin2[G] and Pin2[14] came from the venom of the scorpion *Pandinus imperator* [16,19]. Peptides P18K and FA1 came from the venom of the scorpions *Vaejovis mexicanus* and *Hadrurus gertschi*, respectively [17]. Endoproteinases pepsin, chymotrypsin, elastase and trypsin were from Sigma Aldrich (St. Louis, MO, USA). The antibiotics Levofloxacin, Trimethoprim, Sulfamethoxazole, Azithromycin, Amoxicillin, Ceftriaxone, Gentamicin and Vancomycin were donated from Laboratorios Liomont SA de CV (Mexico City, Mexico).

### 4.2. Peptide Synthesis and Purification

The four peptides were chemically synthesized by a solid-phase method using the Fmoc methodology. The crude synthetic peptides were dissolved in 20% aqueous acetonitrile solution and separated by reverse phase HPLC on a semipreparative C_18_ column (10 × 250 mm, Nacalai Tesque, Japan). The C_18_ column was equilibrated in 20% aqueous acetonitrile containing 0.1% TFA. The synthetic peptides were separated using a linear gradient of acetonitrile/0.1% TFA (from 20% to 60% in 40 min), at a flow rate of 2 mL/min. Effluent absorbance was monitored at 230 nm. Main fractions with antimicrobial activity were finally purified using a reverse phase analytical C_18_ column (4.6 × 250 mm, Vydac 218 TP54, Grace Co., Columbia, MD, USA) using a gradient from 0 to 60% in 60 min at flow rate of 1 mL/min and monitored at 230 nm. The same procedure was used for all four synthetic peptides. The structural identity of each peptide was verified using N-terminal sequencing by automatic Edman degradation, and by mass spectrometry, as indicated below.

### 4.3. Sequence Analysis and Mass Spectrometry

The mass identity of all isolated peptides and the peptide fragments from the enzymatic cleavage was verified by ESI-MS using a Finnigan LCQ^DU^° ion trap mass spectrometer (San Jose, CA, USA). The antimicrobial peptides and their enzymatic fragments were sequenced from their N-terminal by Edman degradation using a LF3000 Protein Sequencer (Beckman, CA, USA). Supplementary Figure S1 shows the mass spectra of the four peptides.

### 4.4. Antimicrobial Activity

It was determined using the broth microdilution assay in accordance with the procedures from the Clinical and Laboratory Standards Institute [20]. For broth microdilution assays, pathogenic bacteria, such as *Escherichia coli* ATCC 25922, *Staphylococcus aureus* ATCC 25923, *Pseudomonas aeruginosa* ATCC 27853 and *Klebsiella pneumoniae* ST258, were cultured in Mueller-Hinton broth (MHB) at 37 °C until an end point between of 0.08 and 0.13 units of absorbance at 625 nm and diluted 1:100 in MHB (*ca.* 1 × 10^8^ CFU/mL). Fifty microliters of this bacterial suspension was dispensed into each well of a 96-well microtiter Costar^®^ culture plates (Sigma Aldrich) having 50 µL of MHB containing a proper concentration of pure AMPs, antibiotics or the mixture of either of the AMPs with either of the antibiotics used. Dilutions of peptides and antibiotics were made manually with two-fold dilutions. The antibacterial activity of each sample was evaluated by measuring absorbance (A_625nm_) after 18 h of incubation time at 37 °C using a Sunrise™ plate reader from Tecan Group Ltd. (San Jose, CA, USA) taking care that evaporation did not occur during the incubation period.

### 4.5. Antibiotic Analysis

*E. coli*, *S. aureus*, *P. aeruginosa* and *K. pneumoniae* were grown in the presence of each of the four AMPs, and in the presence of each of the eight commercial antibiotics, which were Levofloxacin, Trimethoprim, Sulfamethoxazole, Azithromycin, Amoxicillin, Ceftriaxone, Gentamicin and Vancomycin, and in combinations of AMPs and the antibiotics. That is, the antimicrobial capacity of each AMP (50, 25, 12.5 and 6.2 µg/ mL) in combination with each antibiotic agent (50, 25, 12.5, 3.2, 1.6 and 0 µg/ mL) was measured as described above. For evaluation of the effect of the mixtures, the fractional inhibitory concentration (FIC) was calculated for each antibiotic in each combination [9]. The following formulas were used to calculate the FIC index: FIC of AMP = MIC of AMP in combination with Antibiotic/MIC of AMP alone; FIC of Antibiotic = MIC of Antibiotic in combination with AMP/MIC of Antibiotic alone, and FIC index = FIC of AMP + FIC of Antibiotic. Synergy was defined as an FIC index of <0.5. Additive was defined as an FIC index of >0.5 but of <4, and finally antagonism was defined as an FIC index of >4.

### 4.6. Stability of Peptides

#### 4.6.1. Stability in Saline Buffer

The stability of the four synthetic peptides (20 μg) was measured in phosphate buffered saline (PBS) that consisted of NaCl (137 mM), KCl (2.7 mM), Na_2_HPO_4_ (10 mM) and KH_2_PO_4_ (2 mM) at 37 °C for 7 days (pH 7.4). The stability of peptides in PBS was monitored measuring the height of the peptide peak when separated using reverse phase HPLC under the separation conditions mentioned above.

#### 4.6.2. Stability in Human Blood Plasma and in the Supernatant of Bacterial Growth

The four synthetic peptides were subjected to incubation in human blood plasma or in the supernatant of *S. aureus* and *P. aeruginosa* growth. The AMPs were diluted in human blood plasma or in the supernatant of bacterial growth (50 μg/mL) and incubated at 37 °C for 12 h. After peptide extraction, the AMPs were separated by reverse phase HPLC using a C_18_ column (Vydac 218 TP54, Grace Co.) and a linear gradient of acetonitrile in 0.1% aqueous TFA from 0 to 60% in 60 min at a flow rate of 1 mL/min. Effluent absorbance was monitored at 230 nm. Peptide fractions were dried under vacuum, resuspended and analyzed by ESI-MS to confirm their molecular masses.

#### 4.6.3. Stability in the Presence of Enzymes

The four synthetic peptides (50 μg) were subjected to enzymatic hydrolysis using pepsin, chymotrypsin, elastase and trypsin. The experiments were carried out in 20 mM Tris-HCl (pH 8) at 37 °C for 2 h, using a 1:10 (*w*/*w*) enzyme to substrate ratio, except the hydrolysis using pepsin, which was carried out in 10 mM HCl with the same incubation conditions and enzyme to substrate ratio. After enzymatic digestion, the products were fractionated by reverse phase HPLC using a C_18_ column (Vydac 218 TP54, Grace Co.) and a linear gradient of acetonitrile in 0.1% aqueous TFA from 10% to 60% for 50 min at a flow rate of 1 mL/min. Effluent absorbance was monitored at 230 nm. Peptide fractions were dried under vacuum, resuspended and analyzed by ESI-MS.

### 4.7. Statistical Analysis

The last significant difference method was used to determine whether statistically significant differences occurred among the mean values obtained using the software package Prism 4 (GraphPad, Inc., San Diego, CA, USA).

## Figures and Tables

**Table 1:** Physicochemical properties of the synthetic peptides.

Peptide	Amino Acid Sequences	GRAVY	AMF	µH	Q	RT (min)	Molecular Mass (Da)	
							Theoretical	Experimental §
Pin2[G]	FWGALAKGALKLIGSLFSSFSKKD	0.379	0.429	0.465	+3	51.5	2572.0	2572.4
Pin2[14]	FWGLKGLKKFSKKL	−0.357	0.427	0.417	+5	30.5	1680.1	1680.3
P18K	GILKTIKSIASKLKRKAK	−0.328	0.455	0.573	+7	33.5	1983.5	1984.0
FA1	GILKTIKSIASKVANTVQKLKRKAKNAV	−0.171	0.442	0.535	+8	42.0	3008.6	3009.5

GRAVY, Sequence Grand average of hydropathicity, calculated using the Expasy ProtParam tool [5], according to Kyte and Doolittle, 1982 [6]; AMF, Average Molecular Flexibility values were calculated according to Liu *et al.*, 2008 [7]; µH, Hydrophobic Moment was calculated according to Gautier *et al.*, 2008 [8]. Q, Net charge. RT, Retention Time in minutes; (§) Mass Spectrometry, ESI-MS (Finnigan LCQ^DU^° ion trap mass spectrometer, San Jose, CA, USA).

**Table 2:** Minimal inhibitory concentration of peptides and antibiotics against *E. coli*, *P. aureginosa, S. aureus* and *K. pneumoniae*.

Antimicrobials	Minimal Inhibitory Concentrations (µg/mL) ^a^			
	*E. coli*	*P. aureginosa*	*S. aureus*	*K. pneumoniae*
*Peptides*				
Pin2[14]	>50	>50	>50	>50
P18K	>50	>50	>50	>50
Pin2[G]	50	>50	25	>50
FA1	6.25	25	>50	>50
*Antibiotics*				
Amoxicillin	12.5	>50	1.5	>50
Gentamicin	1.5	1.5	1.5	12.5
Vancomycin	>50	>50	1.5	>50
Ceftriaxone	1.5	12.5	1.5	>50
Sulfamethoxazole	>50	>50	>50	>50
Levofloxacin	1.5	1.5	1.5	50
Azitromicin	1.5	12.5	3.1	>50
Trimethoprim	1.5	>50	1.5	>50

^a^ MIC according to data on Figure 2 and Figure 3.

**Table 3:** Best concentration mixtures of peptides and commercial antibiotics tested to inhibit the growth of *Klebsiella pneumoniae*.

Peptide-Antibiotic	Best Concentration of Mixtures ^a^ (MIC, µg/mL)		Peptide ^b^ (MIC, µg/mL)	Antibiotic ^c^ (MIC, µg/mL)	FIC Index ^d^
	Peptide	Antibiotic			
FA1-Amoxicillin	>50	>50	>50	>50	2>
FA1-Azithromycin	25	1.2	>50	>50	<0.524
FA1-Ceftriaxone	25	1.2	>50	>50	<0.524
FA1-Gentamicin	50	3.1	>50	12.5	<1.25
FA1-Levofloxacin	12.5	12.5	>50	50	<0.48
FA1-Sulfamethoxazole	6.25	1.2	>50	>50	<0.15
FA1-Trimethoprim	50	3.1	>50	>50	<1.062
FA1-Vancomycin	12.5	25	>50	>50	<0.75
Pin2[G]-Amoxicillin	>50	>50	>50	>50	2>
Pin2[G]-Azithromycin	25	25	>50	>50	<1
Pin2[G]-Ceftriaxone	50	12.5	>50	>50	<1.25
Pin2[G]-Gentamicin	6.25	12.5	>50	12.5	<1.25
Pin2[G]-Levofloxacin	25	25	>50	50	<1
Pin2[G]-Sulfamethoxazole	50	1.2	>50	>50	<1.024
Pin2[G]-Trimethoprim	>50	>50	>50	>50	2>
Pin2[G]-Vancomycin	50	50	>50	>50	<2

^a^ The best concentration of mixtures are based on the results shown in Figure 4 and Figure 5; ^b,c^ The MICs are based on the results shown in Figure 2 and Figure 3; ^d^ FIC Index; <0.5 synergistic; >0.5 and 4< additive; 4> antagonistic.

## Supplementary Materials

Supplementary materials can be accessed at: http://www.mdpi.com/1420-3049/21/2/225/s1.
