# Supplementary material for: Antimicrobial Activity and Stability of Short and Long Based Arachnid Synthetic Peptides in the Presence of Commercial Antibiotics
Source: Molecules. 2016 Feb 17;21(2):225. doi: 10.3390/molecules21020225 (PMC6273820; doi:10.3390/molecules21020225)
Supplement: Supplementary file 1 [file molecules-21-00225-s001.pdf]

# Supplementary Materials: Antimicrobial Activity and Stability of Short and Long Based Arachnid Synthetic Peptides in Presence of Commercial Antibiotics

**Ivan Arenas, Elba Villegas, Oliver Walls, Humberto Barrios, Ramon Rodríguez and Gerardo Corzo ***

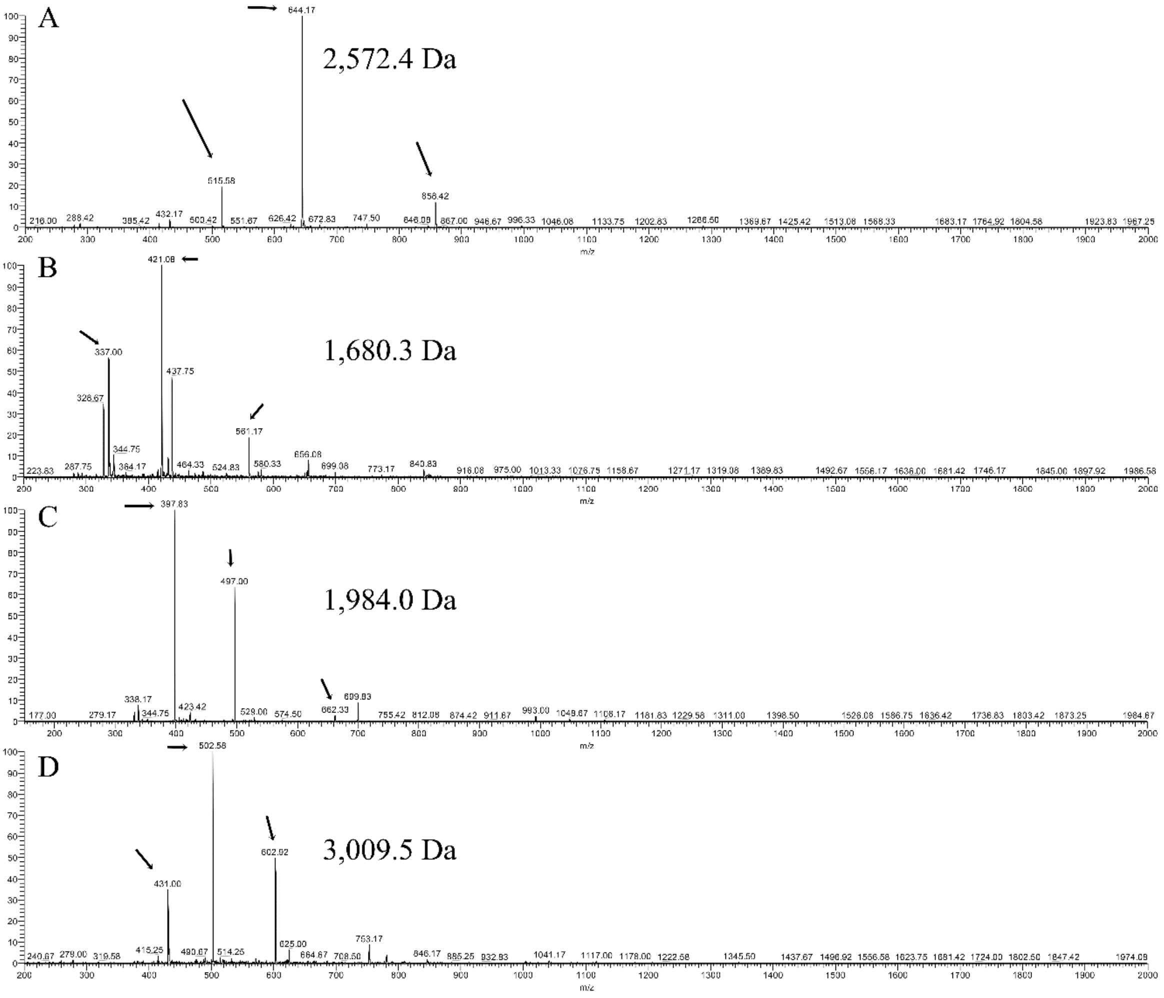


**Figure S1.** Mass spectra of the AMPs. The arrows indicate the main *m*/*z* ions of each peptide. (**A**) Pin2[G]; (**B**) Pin2[14]; (**C**) P18K and (**D**) FA1.
